# Prevalence of Increased QTc Dispersion Among Hypertensive Patients and Its Correlation to Clinical Risk Factors: A Hospital-Based Case-Control Study

**DOI:** 10.7759/cureus.56423

**Published:** 2024-03-18

**Authors:** Daniel Ohemeng Minkah MD FWACP, Isaac K Owusu, Collins Kokuro, Betty R Norman, Joshua A Arthur, ISAAC N Ogyefo, Anthony G Kweki

**Affiliations:** 1 Internal Medicine, Komfo Anokye Teaching Hospital, Kumasi, GHA; 2 Medicine, Kwame Nkrumah University of Science and Technology, Kumasi, GHA; 3 Cardiology, Komfo Anokye Teaching Hospital, Kumasi, GHA; 4 Internal Medicine, Kwame Nkrumah University of Science and Technology, Kumasi, GHA; 5 Epidemiology and Public Health, Ghana Health Service, Accra, GHA; 6 Medicine and Surgery, Kwame Nkrumah University of Science and Technology, Kumasi, GHA; 7 Medicine and Surgery, Komfo Anokye Teaching Hospital, Kumasi, GHA; 8 Internal Medicine and Cardiology, Colchester Hospital, East Suffolk and North Essex NHS Foundation Trust (ESNEFT), Colchester, GBR

**Keywords:** cross-sectional study, prevalence, qtc dispersion, left ventricular mass index, left ventricular hypertrophy, hypertension

## Abstract

Background

In Ghana and other sub-Saharan African countries, hypertension (HTN) prevalence is rapidly increasing. Hypertensive left ventricular hypertrophy (LVH) is associated with excess fibrous tissue deposition throughout the myocardium. This could lead to ventricular arrhythmias and sudden cardiac death. Increased corrected QT dispersion (QTcd) can cause ventricular repolarization and be used to identify patients at risk of ventricular tachyarrhythmia. The measurement of increased QTcd among hypertensive patients is a simple screening tool to stratify patients at cardiovascular risk.

Methods

A case-control hospital-based study was conducted on 200 consecutive hypertensive patients. Age- and sex-matched control groups of 200 normotensive individuals who gave informed consent were also recruited. The baseline clinical and demographic characteristics of participants were acquired using structured questionnaires. A physical examination and a resting 12-lead ECG were performed. Increased QTcd and LVH were determined.

Results

The mean age of hypertensive patients was 50.99±6.73 and 48.19±7.17 for the controls (p-value 0.63). The study population was predominantly female (1:2.4 male:female ratio). Higher mean values for QTcd and LVH (Sokolow-Lyon) were observed among hypertensive patients compared to controls. The prevalence of increased QTcd was 45.0% among hypertensive patients compared to 16.5% in controls (χ^2^ =38.14, p-value <0.0000001, odds ratio = 4.14).

Conclusion

Increased QTcd is prevalent among hypertensive Ghanaians. Its measurement can be an effective non-invasive screening tool to risk-stratify hypertensive patients.

## Introduction

Hypertension (HTN) is one of the leading causes of cardiovascular disease and premature mortality in the world, and its role is set to continue [1]. The global burden of hypertension data from 2005 [2] showed that more than a quarter of the world’s adult population (nearly 1 billion) had HTN in 2000. An increase of about 60% (1.56 billion) is projected in 2025, with the population burden being greater in developing countries like Ghana and Nigeria [3].

In traditional African societies, HTN, once rare, is rapidly becoming a major public health burden [4]. The increasing prevalence of HTN is demonstrated by the increasing mortality from cardiovascular disease (CVD) [5]. Its prevalence is on the increase in developing countries, where the adoption of Western lifestyles and the stress of urbanization are on the rise [6]. Both a poor lifestyle and stress are expected to increase the morbidity associated with HTN [1,2]. Genetic and environmental factors are reported to play a key role in HTN, 90% of which are better classified as idiopathic [7]. Globally, the burden of HTN and other non-communicable diseases (NCDs) is rapidly increasing, and the African continent may be the most affected region in the world [8].

The QT interval measures the duration of ventricular depolarization as well as repolarization; as such, it's an important screening modality that identifies those at risk of CVD. The variability of the QT interval on the leads on the standard ECG with 12 leads is defined as QT dispersion reflecting regional differences in ventricular repolarization [9]. Ventricular repolarization is a complex process occurring non-uniformly in space and time, with the ST segment and T wave on the surface ECG reflecting an integrated signal from multiple repolarization wavefronts [10].

A QT interval prolongation is also essential in predicting sudden cardiac death (SCD) in patients with chronic ischemic heart disease, hypertensive heart disease (HHD), sickle cell anemia patients, and even healthy individuals [11]. The QT interval duration varies between leads on the standard 12-lead ECG. These inter-lead differences, called QT dispersion or QT range, have been proposed as an index of the spatial dispersion of the ventricular recovery times [12]. A corrected QT (QTc) prolongation has been detected as a major cause of morbidity and mortality among hypertensive patients [13].

Prolonged QTc is therefore a predictor of cardiovascular mortality [13]. An increase in QTc dispersion (QTcd) is an electrocardiographic feature of ventricular repolarization as well as a risk marker for ventricular tachyarrhythmia [11]. These potentially dangerous arrhythmias associated with prolonged QTcd and LVH include ventricular couplets, ventricular tachycardia, and ventricular fibrillation [14]. This is thought to be due in part to the induction of pro-arrhythmic repolarization changes caused by the imbalance between the myocytes and the interstitium of the myocardial skeletal structure occurring in pathological LVH [15]. Increased QTcd has been associated with hypertensive subjects in the presence of LVH [16].

If this hypothesis is true, it is therefore expedient that the measurement of QTcd might be a simple, non-invasive screening procedure to identify patients who are hypertensive at Komfo Anokye Teaching Hospital (Kumasi, AH, GHA), who may be at increased risk of ventricular arrhythmias and SCD. Therefore, the need is to focus on how to screen, detect, and treat high-risk patients to prevent cardiovascular mortality and SCD. This study provides relevant data on the increased QTcd that informs the development of local standard management guidelines and protocols for HTN management. The study aims to determine the prevalence of increased QTcd in hypertensive patients and correlate the clinical characteristics of patients with increased QTcd.

## Materials and methods

The study was conducted at the cardiology outpatient clinic of Komfo Anokye Teaching Hospital, a 1200-bed tertiary-level health facility located in Kumasi in the Ashanti region of Ghana. The study is a hospital-based case-control study involving adult patients with HTN aged ≥ 18 years presenting at the cardiology clinic. Age and sex-matched non-HTN or normotensives from the general outpatient department of the family medicine department of the same hospital were recruited as the control group. The data for the study was acquired between January 2020 and March 2021.

Inclusion criteria include consenting adult patients aged 18 years and older diagnosed with essential HTN, on antihypertensives, and not on any other drugs such as antimalarials, digoxin, calcium supplements, amiodarone, and phenothiazine. Subjects with chronic kidney disease (CKD), heart failure, pregnancy, diabetes mellitus, stroke, congenital heart disease, or valvular heart disease were excluded from the study, as increased QTc may also be seen in these conditions. More so, subjects who had taken medications such as antidepressants, antipsychotics, digoxin, and antibiotics such as erythromycin or any drug that is likely to prolong the QT interval in the week before recruitment were also excluded. Ethical approval was obtained from the Committee on Human Research, Publication, and Ethics, School of Medical Sciences, Komfo Anokye Teaching Hospital (approval no. CHRPE/AP/624/19).

A total of 200 cases and 200 control subjects were recruited through simple random sampling. A standard conventional 12-lead resting ECG was recorded at 25 mm/s and 1 mV/cm standardization using the Advanced® ECG-3 Plus machine (Advanced Instrumentations, Miami, FL, USA) for all the participants. With the subject relaxed and comfortably lying supine, the electrodes were placed, and effective skin contact was ensured. It was regularly checked for technical faults such as damping and electrical interference. The ECG tracings were analyzed and interpreted using calipers. The ECGs were analyzed quantitatively to obtain heart rate, rhythm, QRS axis, P wave, QRS morphology, PR interval, and QRS morphology. The QT intervals in each of the leads were measured. The LVH was determined using the Sokolow-Lyon criteria. The QT interval (QT) was then manually measured in all 12 leads of the ECG and corrected for heart rate using the formula of Hodges et al. (QTc = QT + 1.75 (ventricular rate - 60) [17]. The QTcd defined as QT dispersion (QTd) max - QTd min, was then calculated. Adjusted QTd was measured to correct for the known dependence of the index on the number of measured leads. The QTc prolongation was defined as QTc≥440 ms, while increased QTcd was determined to be QTcd ≥ 80 ms [12].

The data was analyzed using SPSS Statistics version 25.0 (IBM Corp., Armonk, NY, USA). The mean± SD was used in reporting quantitative variables, while non-quantitative variables were presented as percentages. Chi-square and Student's t-tests were used, where applicable, to compare quantitative variables among subjects. Pearson's correlation coefficient analysis was performed, and variables that show a significant positive relationship to QTd were then entered into a binary logistic regression analysis. A p-value <0.05 was considered as the level of statistical significance in each case.

## Results

Sociodemographic characteristics of participants

The sociodemographic characteristics of participants are represented in Table 1. There were 59 (29.5%) male and 141 (70.5%) female hypertensive patients, 53 (26.5%) male, and 147 (73.5%) female non-hypertensive patients. The mean age of hypertensive patients was 50.99±6.78 and that of controls was 48.19±7.17; there was no significant statistical difference between the mean ages of both groups (p-value = 0.630). Most of the 140 (70.0%) hypertensive patients were married, while 17 (8.5%) were single, 13 (6.5%) were divorced, and 30 (15.5%) were widowed. For the controls, the majority of 150 (75.5%) were married, 25 (12.6%) were single, four (2.0%) were divorced, and 19 (9.6%) were widowed. As for the educational level of hypertensive patients, 97 (48.5%) had a primary level of education, 46 (23.0%) reached the secondary level, 30 (15.0%) were university-level, 25 (12.5%) had no formal education, and two (1.0%) had technical education. For the controls, a majority of 107 (53.5%) had a secondary level education, 33 (16.5%) had a primary level education, 24 (12.0%) had no formal education, 32 (16.0%) had reached university level, and four (2.0%) had technical education. For the hypertensive patients, the majority were Christians, i.e., 175 (87.5%), and 25 (12.6%) were Muslims. For the controls, the majority of 149 (74.5%) were Christians, 49 (24.5%) were Muslims, and two (1.0%) were of another religion. Most of the hypertensive patients were employed (164 (82.0%)), 35 (15.5%) were unemployed, and one (0.5%) was a student. For the controls, the majority (163 (74.5%)) were employed, 33 (16.5%) were unemployed, and four (2.0%) were students.

**Table 1:** Sociodemographic characteristics of participants

Variables		Hypertensive subjects		Control subjects	
		Frequency	Percentage	Frequency	Percentage
Sex	Female	141	70.5	147	73.5
	Male	59	29.5	53	26.5
Religion	Christianity	175	87.5	149	74.5
	Islamic	25	12.6	49	24.5
	Others	0	0.0	2	1.0
Marital status	Married	140	70.0	150	75.8
	Single	17	8.5	25	12.6
	Divorced	13	6.50	4	2.0
	Widowed	30	15.0	19	9.6
Occupation	Employed	164	82.0	163	81.50
	Unemployed	35	15.5	33	16.50
	Student	1	0.5	4	2.00
Education	No formal education	25	12.5	24	12.0
	Primary	97	48.5	33	16.5
	Secondary	46	23.0	107	53.5
	Technical	2	1.0	4	2.0
	University	30	15.0	32	16.0

Clinical characteristics of participants

The clinical characteristics of the study participants, such as age, BMI, waist-to-hip ratio, blood pressure, and heart rate, are shown in Table 2. The mean age of hypertensive subjects was 50.99±6.78 compared with the mean age of control subjects (48.19±7.17, p-value 0.630). The BMI, waist-to-hip ratio, blood pressure, and heart rate were significantly higher among hypertensive subjects than among controls.

**Table 2:** Clinical characteristics of participants WHR: Waist to hip ratio, SBP: Systolic blood pressure, DBP: Diastolic blood pressure

Variables	Hypertensive subjects	Control subjects	p-value
	Mean ± SD	Mean ± SD	
Age (years)	50.99±6.78	48.19±7.17	0.630
BMI (kg/m^2^)	28.61±5.91	24.91±2.42	0.010
WHR	0.94±0.08	0.89±0.04	0.001
Mean SBP (mmHg)	153.97±23.52	121.26±6.69	0.001
Mean DBP (mmHg)	90.91±14.98	79.5±7.04	0.014
Heart rate (beat/min)	86.13±14.84	75.57±10.42	0.010

ECG characteristics of participants

**Figure 1:**
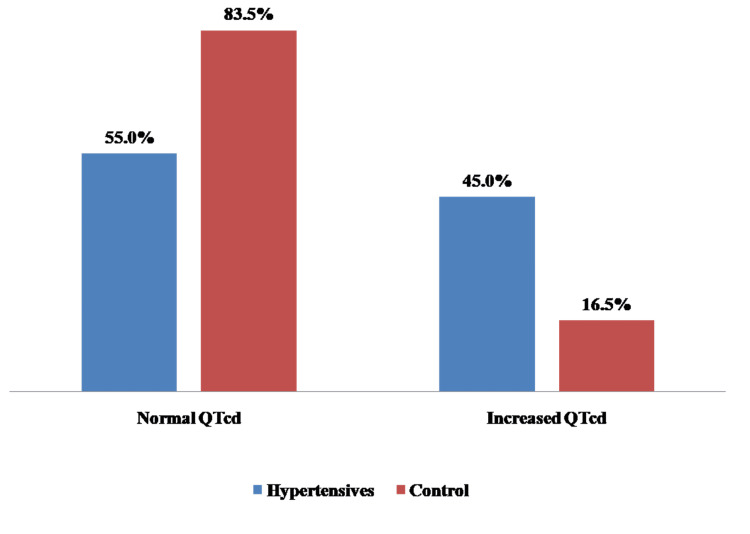
Prevalence of increased QTcd χ^2^ =38.14, p-value <0.0000001, odds ratio=4.14 QTcd: Corrected QT dispersion

Electrocardiography was performed on both hypertensive and control subjects to study relevant characteristics. The results are presented in Table 3 below. Among hypertensive patients, higher mean values were recorded for QTcd (66.10 vs. 49.92) and LVH Sokolow-Lyon (31.07 vs. 23.23). The prevalence of increased QTcd was 45.0% among hypertensive patients compared to 16.5% in controls (χ2 =38.14, p-value <0.0000001, odds ratio=4.14) (Figure 1).

**Table 3:** ECG characteristics of the hypertensive patients QTcd: Corrected QT dispersion, LVH: Left ventricular hypertrophy

ECG characteristics	Hypertensive	Control	p-value
	Mean ± SD	Mean ± SD	
QTcd	66.10±24.94	46.92±21.53	0.023
LVH Sokolow-Lyon	31.07±11.02	23.23±4.24	0.0039

Cardiovascular risk factors associated with increased QTcd and LVH

Some commonly known cardiovascular risk factors associated with increased QTcd and LVH among hypertensive patients were assessed. A total of 277 respondents without increased QTcd and LVH were compared with 85 respondents who had both increased QTcd and LVH. The significant associations were age, family history of HTN, smoking, and physical inactivity. Of note, BMI, family history of diabetes mellitus, and family history of hyperlipidemia did not show a significant association with increased QTcd and LVH in the study population. (Table 4). The binary logistic regression is represented in Table 5.

**Table 4:** Cardiovascular risk factors associated with increased QTcd and LVH * Significance (p-value <0.05) QTcd: Corrected QT dispersion, LVH: Left ventricular hypertrophy, HTN: Hypertension

Variables	Category	Without increased QTcd and LVH	With increased QTcd and LVH	χ^2^
Age	30-40	35 (12.7)	5 (5.9)	6.69*
	41-50	120 (43.3)	30 (35.3)	
	>50	122 (44.0)	50 (58.8)	
Sex	Female	209 (75.5)	58 (68.2)	1.75
	Male	68 (24.5)	27 (31.8)	
Family history of HTN	No	161 (58.1)	23 (27.1)	25.11*
	Yes	116 (41.9)	62 (72.9)	
Family history of diabetes mellitus	No	239 (86.3)	77 (90.6)	1.09
	Yes	38 (13.7)	8 (9.4)	
Family history of hyperlipidemia	No	276 (99.6)	85 (100.0)	0.31
	Yes	1 (0.4)	0 (0.0)	
Smoking	No	277 (100.0)	83 (97.7)	6.55*
	Yes	0 (0.0)	2 (2.3)	
Physical inactivity	Yes	204 (73.7)	83 (97.7)	22.81*
	No	73 (26.4)	2 (2.3)	
BMI	Normal	108 (39.0)	32 (37.7)	0.05
	Obese/overweight	169 (61.0)	53 (62.4)	

**Table 5:** Binary logistic regression Number of subjects observed = 362, LR chi2(14) = 143.80, Prob > chi2 = 0.001, Pseudo R2 = 0.3644, Log-likelihood = -125.4 QTcd: Corrected QT dispersion, DM: Diabetes mellitus, HTN: Hyperstension

Increased QTcd	Crude				Adjusted			
	OR	z	P>z	95% CI	OR	z	P>z	95% CI
Age 41 to 50 years	1.75	1.08	0.282	0.63-4.85	2.119	1.04	0.298	0.51-8.72
Age > 50 years	2.868	2.08	0.038	1.06-7.75	2.842	1.46	0.144	0.70-11.56
Family history of HTN	3.741	4.84	0.000	2.19-6.39	1.605	1.27	0.205	0.77-3.33
Family history of DM	0.653	-1.04	0.300	0.29-1.46				
Physical inactivity	0.067	-3.70	0.000	0.02-0.28	0.167	-2.23	0.026	0.03-0.81
BMI	1.058	0.22	0.824	0.64-1.75				
Systolic	1.046	7.57	0.000	1.03-1.06	1.025	2.44	0.015	1.00-1.05
Diastolic	1.052	5.21	0.000	1.03-1.07	1.003	0.20	0.844	0.97-1.04
Heart rate	1.028	3.16	0.002	1.01-1.05	0.997	-0.28	0.778	0.97-1.02

## Discussion

To our knowledge, this is the first study to assess the pattern and clinical correlates of QTcd among Ghanaian hypertensive patients. In our sub-region, most studies on QTcd focus on newly diagnosed hypertensive patients, but this study focused on all hypertensive subjects in the cardiology clinic. Similar studies have been done in Nigeria, where QTcd was assessed in newly diagnosed HTN patients [11,12]. The QTcd has become an important non-invasive screening tool to risk-stratify hypertensive patients with increased risk for cardiovascular accidents (CVA) and, therefore, could be used for the reduction of cardiovascular morbidity and mortality in the Ghanaian population.

Sociodemographic characteristics of the study population

Hypertensive subjects with increased QTcd in this study were of similar age to those in the controls. Similar sociodemographic characteristics were found in studies among Nigerians in sub-Saharan Africa [12] and Caucasians [18]. The study population was predominantly female. There were 141 (70.5%) females compared to 59 (29.5%) males among hypertensive patients. For the controls, there were 147 (73.5%) females compared to 53 (26.5%) males. This may probably be because the study was hospital-based and females may have better health-seeking behavior than males [19].

Prevalence of increased QTcd among hypertensive patients

The findings of the index study revealed that a significant proportion of hypertensive Ghanaians seen in the cardiology clinic are already at increased cardiovascular risk because increased QTcd is associated with increased cardiovascular risk. The prevalence of increased QTcd (QTcd ≥ 80 ms) in this study was 45.0% in the hypertensive patients and 16.5% among controls. This is comparable to the report by Familoni et al. among subjects with acute ischaemic stroke [13].

Another study in Nigeria also reported that the prevalence of increased QTcd ≥ 80 ms was 36.43% among hypertensive subjects, while only 18.57% of the control group had increased QTcd [12]. The frequency of occurrence of increased QTcd ≥ 80 ms was, however, greater in this study than that by Akintunde et al. [12] and Familoni et al. [13], who reported 36.43% and 18.57% prevalence among hypertensive and controls, respectively, in the south-western part of Nigeria. The difference in prevalence could be because the study done by Akintunde et al. [12] recruited newly diagnosed hypertensive patients, while this study recruited both newly diagnosed and hypertensive patients already on treatment.

Increased QTcd (QTcd ≥ 80 ms) is a good predictor of arrhythmia and sudden cardiac death (SCD) in such conditions as long QT syndrome, patients with hypertrophic cardiomyopathy, chronic heart failure, post-myocardial infarction patients, HTN, and diabetes mellitus patients [20]. Some studies have suggested that prolonged QTcd is an independent and stronger risk factor for cardiac mortality in the elderly, stronger than LVH and systemic HTN [14].

In addition, this study found that 16.5% of our controls have an increased QTcd (> 80 ms). The control group had normal blood pressure, left ventricular mass, and no evidence of LVH from the ECG. This observation may therefore mean that either there is congenital long-QT interval syndrome within the population studied or we are seeing the effect of drugs that might have pro-arrhythmic effects not volunteered by the participants. The congenital long-QT is highly unlikely because none had a history of recurrent syncope, a family history of childhood SCD, or congenital deafness. The QTcd value was far less than that reported for patients with congenital long QT syndrome (>100 ms) [20]. The effect of drugs may likely explain our findings because getting correct and accurate information can be difficult in our local communities. This may be partly due to ignorance. These controls may have taken some drugs known to prolong QT intervals, such as antimalarials, antibiotics, and antihistamines. However, in Ghana, there is a lack of testing for serum levels before starting a study, which could have helped with the problem. Even though the occurrence of drug-induced torsade-de-pointes is low, there is a growing list of drugs that can cause acquired long-QT syndrome, which may pose a real concern to our Ghanaian community [21]. In addition to this, there may also be non-clinical viral myocarditis and rheumatic fever with attendant fibrosis, resulting in prolonged QTcd among the controls. The fact that 45.0% of hypertensive subjects had increased QTcd, which is a noninvasive stratification tool, highlights the enormous role of early implementation of total cardiovascular care, including adequate stratification and management for hypertensive subjects.

Cardiovascular risk factors associated with increased QTcd and LVH

The study revealed that commonly known cardiovascular risk factors associated with increased QTcd and LVH among hypertensive patients were age, family history of HTN, smoking, and physical inactivity. Of note, BMI, family history of diabetes mellitus, and family history of hyperlipidemia did not show a significant association with increased QTcd and LVH in the study population.

According to the study, as the age of the study population increases, there is a significant increase in the QTcd and all the associated cardiovascular risks. Prolonged QTc intervals and increased QTcd in the elderly are associated with a threefold increased risk of SCD, mostly due to ventricular tachyarrhythmias [22]. The age range of patients with significant associations was 41 to 50 years and above 50 years. The study corroborates an earlier report by Haider et al. [21].

The study also shows a significant measure of association between increased QTcd with LVH and a positive family history of HTN (χ2 =25.11). The family history of HTN was for first-degree relatives or parents, and this affirms the fact that there are genetic factors associated with HTN and other cardiovascular risks. The family history of HTN is a risk factor for HTN. Therefore, a family history of HTN has an indirect effect on increased QTcd and LVH.

There have been contradictions regarding the effects of smoking on QTc and QTcd. While Taşolar et al. [23] did not identify any effects of smoking status on QTc, Dilaveris et al. [24] observed significantly lower values of QTc in smokers without differences in QTcd. A study that also assessed the dispersion of repolarization showed significantly higher values of QTc and QTcd in non-smokers [23]. This study identified a higher measure of association between non-smoking and increased QTcd. The majority of the study population were non-smokers, and smoking cigarettes may not be as prevalent in Ghana as it is in the Western world.

Physical inactivity is a known risk factor for HTN, diabetes mellitus, and other cardiovascular diseases. This study revealed that most of the participants with HTN did not meet the recommended level for physical activity by the WHO, which requires adults aged between 18 and 64 years to accumulate at least 150 minutes of moderate-intensity aerobic physical activity throughout the week. Alternatively, undertake at least 75 minutes of vigorous-intensity aerobic physical activity throughout the week, or a combination of both [25]. The study showed a significant measure of association between physical inactivity and increased QTcd (χ2=22.81). The association may probably be indirect. 

Numerous studies suggest that obesity, particularly the central type of obesity, is associated with delayed ventricular repolarization that leads to a prolonged QTc interval and QTcd [26]. One of the causes of QTc prolongation may be an imbalance of the autonomic nervous system in obesity [26]. Although obesity is commonly associated with increased QTcd and increased cardiovascular risk, this study did not reveal any significant association between BMI and increased QTcd (χ2 = 0.05). The overweight and obese were categorized together, but there was no significant association. The study did not have a higher number of obese patients, so the measure of association was low.

Diabetes mellitus and hyperlipidemia are important known risk factors also associated with cardiovascular disease and increased QTcd [15]. However, this study excluded patients with diabetes mellitus, and the fasting lipids of participants were not measured because of budgetary constraints. Family history of diabetes mellitus and hyperlipidemia were assessed among participants, but the measure of association was low (χ2 = 1.09 and 0.31, respectively). This might probably be because most people in our community may not know much about hyperlipidemia and diabetes mellitus as compared to HTN, which is under-reported.

Limitations

The study has several limitations. First, there is a lack of a prospective arm of the study, which makes a categorical statement on the reduction of morbidity and mortality very weak. Another limitation noted was that other causes of QT prolongations, such as congenital QT syndromes and acquired QT prolongations, were not measured in this study. Lastly, this study measured QTcd manually and may contain all the method's inherent inter- and intra-observer defects. Using an automated computer algorithm with a digital board would have been more accurate for the determination of QTcd. These facilities are lacking in Ghana. Therefore, until our government makes provisions for automation, we believe that the manually calculated QTcd may still be a simple, affordable, and noninvasive method for the detection of hypertensive subjects with an increased risk of SCD.

## Conclusions

Increased QTcd is prevalent among hypertensive Ghanaian subjects attending the cardiology clinic. Cardiovascular risks associated with increased QTcd and LVH included age, family history of HTN, smoking, and physical inactivity. Increased QTcd can be used as a non-invasive marker of HTN with increased cardiovascular risk and can therefore be a surrogate screening tool for lowering the risk of heart disease and death in the population. However, BMI, family history of diabetes mellitus, and family history of hyperlipidemia did not show a significant measure of association.
